# Waveguide effective plasmonics with structure dispersion

**DOI:** 10.1515/nanoph-2021-0613

**Published:** 2021-12-08

**Authors:** Xu Qin, Wangyu Sun, Ziheng Zhou, Pengyu Fu, Hao Li, Yue Li

**Affiliations:** Department of Electronic Engineering, Tsinghua University, Beijing 100084, China; Beijing National Research Center for Information Science and Technology, Tsinghua University, Beijing 100084, China

**Keywords:** localized surface plasmon, plasmonics, structural dispersion, surface plasmon polaritons, waveguide effective plasmonics

## Abstract

Plasmonic phenomena on the surface between metal and dielectric have received extensive attention, and have boosted a series of exciting techniques. Plasmonics describes the interaction between light and electronics and shows great potential in nanophotonics, optoelectronic devices, quantum physics, and surface-enhanced spectroscopy, etc. However, plasmonic phenomena are always suffering from the inherent loss issue of plasmonic materials at optical frequency, which has restricted further applications of plasmonics. In this review, we focus on the technique of waveguide effective plasmonics, which is a feasible low-loss realization of plasmonic metamaterials in lower frequency based on the structural dispersion. This review provides the underlying physics of the waveguide effective plasmonics and its applications varying from classical plasmonic concepts to novel effective plasmonic devices. Finally, we make a brief discussion on the direction of future researches and a prospect of the potential applications.

## 1 Introduction

Since Mie and Ritchie first systematically described the fundamental physics on the interfaces between conductors and insulators for small particles and flat films, respectively [1, 2], various researches on the surface electron oscillations have been widely conducted, including the propagating electromagnetic wave coupled to the conductor and dielectric interfaces, and the non-propagating resonance in the subwavelength nanostructure coupled to electromagnetic fields. Such surface-confined electromagnetic fields have been investigated as the surface plasmon polariton (SPP) and the localized surface plasmon (LSP), which are two basic components of plasmonics [3].

As the enhanced Raman scattering was discovered by Albrecht [4] and Jeanmaire [5], light-matter interaction between the Raman scattering and the nanostructure in the metal surface has been revealed. It is widely acknowledged that the intensely localized oscillations in regions between nanoparticles are responsible for the enhancement of the Raman scattering, and this phenomenon is known as surface-enhanced Raman scattering [6]. Due to the large enhancement factor and compact dimensions, the surface-enhanced spectroscopy has attracted wide attention and investigations [7], [8], [9], meanwhile, the researches on plasmonics also got developed with the progress in surface-enhanced Raman scattering.

More recently, a large variety of applications of surface plasmons have been developed based on the rapidly developing nanofabrication techniques, such as self-assembly, ion-beam milling, etc. [10, 11]. These applications have exhibited promising abilities of light concentrations and manipulations in dimension-miniaturized optical devices, showing great potential in diverse fields. Researches on these fields would lead to a boost in a new type of devices or even a new discipline, Atwater named this new discipline combining photonics and electronics as “Plasmonics” [12].

Despite the booms of researches on plasmonics, this field is traditionally suffering from non-negligible loss, which has restricted the further progress in practical applications of plasmonics [13]. Noble metals such as gold and silver are traditional plasmonic materials, which have almost the lowest loss in the range of metals. However, at optical frequency, the loss is still remarkable due to the inherent dissipations and absorptions. Therefore, in a long period, the surface-enhanced Raman scattering is a main efficient application for plasmonics as the absolute efficiency is typically low [14]. Afterwards, many attempts have been made in order to seek low-loss plasmonic materials with acceptable optical performance, such as doped-semiconductor, transparent conducting oxide, metal alloys, and graphene, etc. Those studies have demonstrated properties of the low-loss plasmonic materials in near-infrared and visible ranges for a variety of application fields, providing a guideline for material choice [15, 16].

On the other hand, Pendry [17] and Garcia-Vidal [18] first proposed new structures of metamaterials that mimic the property of plasmonic materials, thus broadening the concept of plasmonics. Generally, by introducing periodical structures, such as grooves, holes, slits, and blocks, the SPP-like dispersion relationship can be achieved in the artificial materials. This new concept of plasmonic metamaterial is named spoof SPPs or design SPPs [19]. The spoof SPPs succeeds in constructing plasmonic metamaterials in microwave and terahertz frequencies, while the typical frequency of traditional plasmons is visible and near-infrared ranges, and also provides a tunable and low-loss realization for practical applications of SPP and LSP [20, 21].

Recently, another methodology of constructing plasmonic material is proposed [22]. Based on the structural dispersion of the guided wave propagations, e.g., the parallel-plate waveguides or rectangle waveguides, it is possible to define an effective permittivity, and negative permittivity could be obtained, which is exactly mimicking the property of plasmonic materials [23, 24]. Therefore, the concept of waveguide effective plasmonics is studied as an alternative way of plasmonics in microwave and terahertz regions. Depending on the structure geometry, interior material, and operating frequency, the effective permittivity is tunable from positive to negative, including the special value of zero, of course. Meanwhile, the waveguide effective plasmonics with low-loss and non-dispersive dielectric can avoid the serious loss problem in traditional plasmonic materials. Benefited from low-loss performance and simple structure, waveguide effective plasmonics have earned much attention and have been adopted for various functions, including efficient plasmonic applications operating in microwave frequency [25, 26] with the exciting property of plasmonics [27, 28].

In this review, we aim to systematically demonstrate the fundamental theory of waveguide effective plasmonics based on the waveguide dispersion property, summarizing the design principle and key features of the waveguide effective plasmonics. In the following sections, we will exhibit the applications over a wide range of fields on this simple and low-loss platform, including classical plasmonic concepts, irregular material response, and novel microwave devices with plasmonics. Finally, we close in the conclusions by briefly presenting an outlook of the development for the concept of waveguide effective plasmonics.

## 2 Concept of the waveguide effective plasmonics

### 2.1 Theory

As mentioned above, besides the natural plasmonic material in optical frequency and the artificial periodical metastructure, plasmonic material can be practically realized based on the dispersion in the waveguide with the low-loss property. In this section, we introduce the concept and demonstrate the physical property of the waveguide effective plasmonics.

**Figure 1:**
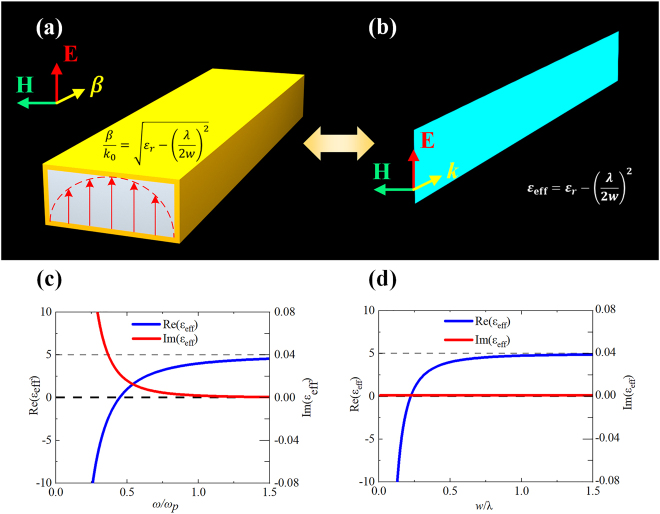
(a) TE_10_ mode in rectangle waveguide; (b) equivalent TEM wave on the middle plane of the waveguide; (c) permittivity of Ag near the plasmonic frequency; (d) effective permittivity of the TE_10_ mode varying with width of the waveguide.

Early in the 1960s, Rotman proved that the parallel-plate or rectangle waveguide can imitate plasmonic materials when the waveguide is operating below the cut-off frequency of TE_10_ mode [23]. Here, we take TE_10_ mode in a rectangle waveguide as an example for single-mode operation. The rectangle waveguide is with the width of *w* and interior dielectric with permittivity of *ε*_*r*_, as illustrated in Figure 1. Due to the transverse resonance, it is known that the TE_10_ mode propagating in the rectangle waveguide has a propagating constant *β* = *k*_0_ (*ε*_r_ − (*λ*/2*w*)^2^)^1/2^, where *λ* is the wavelength and *k*_0_ is the wavenumber in the free space at the operating frequency. Considering the plane wave or transverse electromagnetic (TEM) wave propagating in the dielectric with permittivity of *ε*, the propagating constant is *β* = *k*_0_(*ε*)^1/2^. Therefore, it is reasonable to define the effective permittivity in the TE_10_ mode of rectangle waveguide as *ε*_eff_ = *ε*_*r*_ − (*λ*/2*w*)^2^, such that the effective permittivity *ε*_eff_ reflects the effective property of TE_10_ mode in the rectangle waveguide. On the middle plane of the rectangle waveguide, the wave propagation is the same as the two-dimensional (2D) uniform plane wave propagating in a homogenous dielectric with the permittivity of *ε*_eff_. Therefore, this 2D virtual TEM wave is a representation of the actual TE_10_ mode, as depicted in Figure 1b. The effective permittivity is corresponding to the propagating constant in the waveguide, and specifically, the effective permittivity is positive or negative when the waveguide is operating in propagating or below cut-off mode, respectively, and the effective permittivity equals zero when the waveguide is exactly at the cut-off frequency. Based on this principle, the effective permittivity can vary from positive to negative, and perform the negative index of permittivity to imitate the traditional plasmons at optical frequency. In fact, the effective permittivity in TE_10_ mode has a similar dispersion with the permittivity of plasmonic materials at optical frequency. In Figure 1c and d, the dispersions are depicted, from which we can observe the similarity between the two dispersions. For the rectangle waveguide, the filling dielectric is with the permittivity of 5 + 0.001j, while for the plasmonic material, such as Ag, for example, is with the Drude model *ε*(*ω*) = *ε*_∞_ − *ω*_p_^*2*^/*ω*(*ω* + i*γ*), where *ε*_∞_ is the permittivity in infinity high frequency, *ω*_p_ is the plasma frequency, and *γ* is the collision frequency [29]. We can see the dispersion curves are of the same tendency. Therefore, the TE_10_ mode in rectangle waveguide is a feasible analogy to actual plasmonic materials. Parallel-plate waveguide operates based on the same mechanism as rectangle waveguide of TE mode to realize effective permittivity. The only difference is that the TE modes and TM modes are degenerate modes at the same frequency in the parallel-plate waveguide. When we focus on the effective permittivity, vertical through vias between the plates of the parallel-plate waveguide are usually adopted to suppress TM modes, which in fact account for effective permeability. It is noted that though the fundamental TE_10_ mode is taken as the example, other modes in rectangle waveguide can also realize effective plasmonic materials at appropriate conditions, and even further, the propagating is not limited in rectangle waveguide, any propagating modes with transverse resonance would have the potential for effective plasmonics. When higher modes come into consideration, the orthogonal multi modes operate independently with their effective plasmonic property, and we can extract their property for every single mode. Higher modes could be excited especially by designed feeds if required. However, from a practical perspective, the TE_10_ mode of rectangle waveguide still has its unique superiority for single-mode operation, compact dimension, and simple structure to achieve waveguide effective plasmonics.

Compared with the natural plasmonic materials, the waveguide effective plasmonics features the merits of tunable-frequency and low-loss. The effective permittivity is obtained based on the structural dispersion. By properly choosing the width of the waveguide, the effective permittivity can be easily tuned at a required frequency, which gives more freedom in the operating frequency for plasmonics. In high frequency, the metals no longer provide the boundary of the perfect electric conductor (PEC). As an alternative, effective PEC like photonic crystals could provide the boundary of waveguides to realize the effective plasmonics in high frequency [30]. On the other hand, plasmonics has been suffering from the inherent loss in the natural materials at optical frequencies. However, the waveguide effective plasmonics is able to work with regular dielectric, which has normal non-dispersive positive permittivity and low loss, as the effective permittivity can be tuned by the structural parameters. In this methodology, the intrinsic loss in the plasmonic material is avoidable. On the other hand, by manipulating the effective material parameters with a participation of a parallel-plate waveguide, there is less SPP field distributed in the lossy plasmonic medium, therefore the SPP mode can operate with smaller losses and result in a longer propagation distance [29]. Another methodology, spoof SPPs or design SPPs, also displays the similar property of tunable frequency and reduced loss. And benefit from the low loss in normal material, the spoof SPPs and waveguide effective SPP show similar propagating performance, which is much enhanced than the real SPP, and would be comparable to existing transmission lines, such as rectangle waveguide in propagating mode. The difference with spoof SPPs is the realization mechanism of the plasmonic effect. Generally, the spoof SPPs is constructed with the periodical subwavelength elements. These elements are the microscopic cells for metamaterials, and are an analogy to the atoms in the natural materials, to some extent. Subwavelength dimensions of resonant cells and their massive distributions enable the emulation of nearly uniform plasmonic materials. While in the waveguide effective plasmonics, the dispersion is based on the transverse resonance without periodic resonant cells, which constructs a uniform plasmonic material along the propagating direction.

These natures of the waveguide effective plasmonics can realize high-efficiency plasmonic devices. The applications of plasmonics have been broadened and the design freedom is enhanced. Since the waveguide effective plasmonics is proposed, this methodology has been applied in various fields. Moreover, substrate integrated waveguide (SIW) has been widely adopted in integrated systems as an equivalent configuration of normal rectangle waveguide. SIW is with the same operating mechanism as a normal rectangle waveguide but is highly compatible with the printed circuit board (PCB) technique. Based on the SIW, various applications of waveguide effective plasmonics are highly developed for massive production.

### 2.2 Irregular material response

To manipulate the wave–matter interaction is of key interests to photonics and material sciences. The responses of most common materials to electromagnetic waves are almost linear, time-invariant, reciprocal, and isotropic. On the other hand, the irregular material response to waves, including nonlinearity, nonreciprocity, and anisotropy, have offered opportunities for various extraordinary manipulations of waves, such as ultrafast modulation, asymmetric transmission, and scattering of waves, etc. In most cases, those irregular responses of materials are extremely weak, which thereby limits the efficiency of corresponding processes of wave manipulation. Generally, there are two schemes to enhance the irregular responses of electromagnetic media. One is to enhance the local field intensity, that is, to increase the intensity of external excitation. Another scheme is to exploit artificial structures to build composite materials, e.g., the concept of metamaterials, whose response functions to electromagnetic waves are allowed to be designed, rather than being determined exclusively by the material properties. Plasmonic materials have the ability to localize light in deep subwavelength-scale regions with strongly enhanced fields [31], which benefits the boost of the extremely weak optical processes, such as Raman scattering [8], Fano resonances [32], [33], [34], optical fluorescence [35], [36], [37], [38], and quantum optical effects [39]. For the waveguide-emulated plasmonic materials, their overall dispersion characteristics and local field property can be engineered to attain a significant enhancement of irregular wave responses.

**Figure 2:**
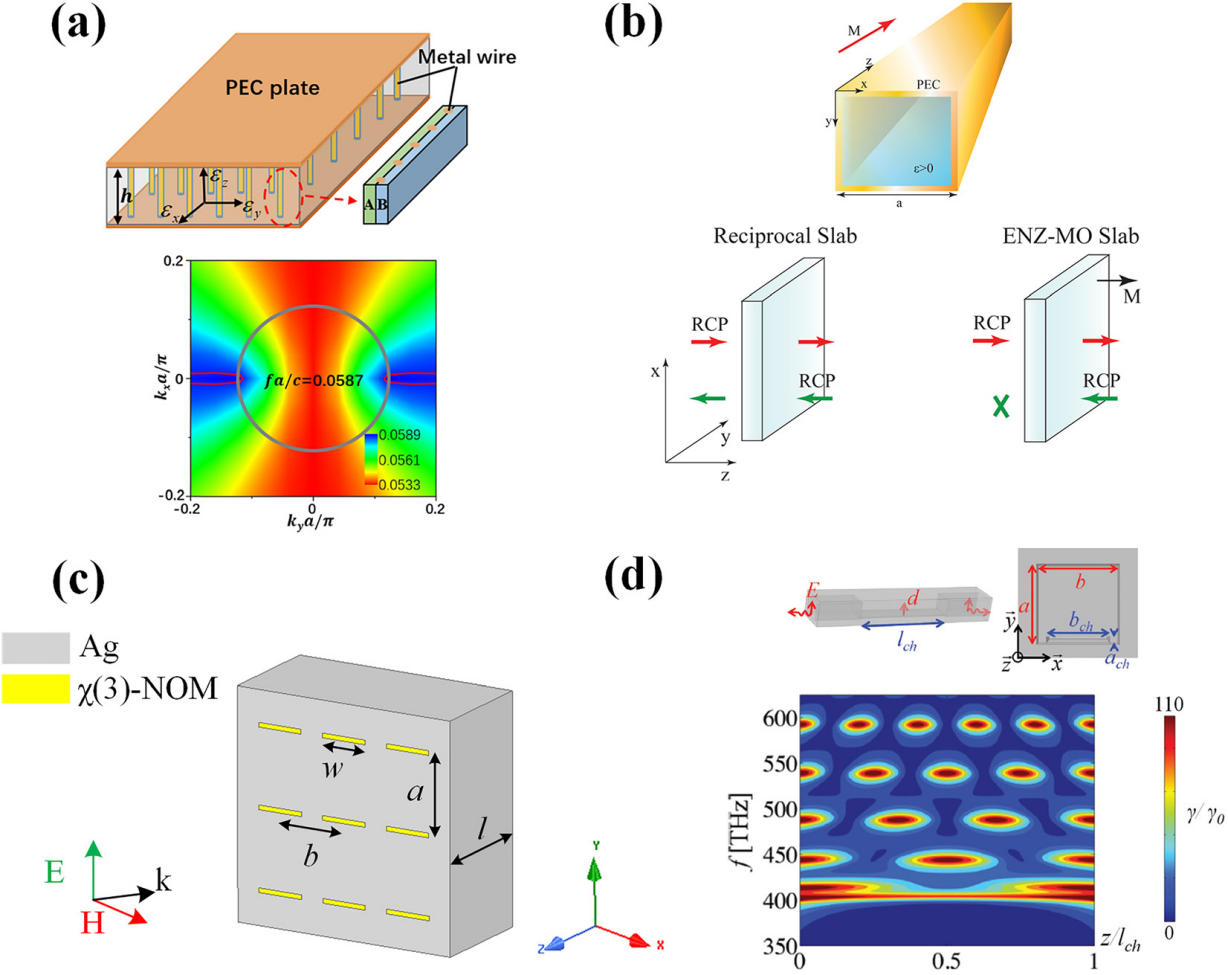
(a) Anisotropic media in waveguide metamaterials [40]; reproduced with permission. Copyright 2019, Optical Society of America. (b) Optical isolator based on non-reciprocal medium [44]; reproduced with permission. Copyright 2013, Optical Society of America. (c) Boosting optical nonlinearity in ENZ channel [45]; (d) quantum emission assisted by ENZ metamaterial [48]; reproduced with permission. Copyright 2013, American Physical Society.

Anisotropy refers to the property of a material exhibiting directionally dependent features, which yields the wave effects such as double refraction or birefringence. Anisotropy can be observed in crystal materials with asymmetric lattices; however, attaining strong anisotropic responses to electromagnetic waves in the optical region is still a challenge. The seminal works by Ji et al. [40, 41] presented a solution to realize extreme anisotropy via a waveguide-emulated plasmonic medium operating on the epsilon-near-zero condition. The top panel of Figure 2a shows the structure of a dielectric layered medium sandwiched by two parallel plates, which is equivalent to a two-dimensional homogenous anisotropic medium on the *x*–*y* plane with its dispersion relationship given by *k*_*x*_^2^/*ε*_*y*,eff_ + *k*_*y*_^2^/*ε*_*x*,eff_ = *k*_0_^2^ in the principal axes system. Here, *k*_*x*_ and *k*_*y*_ are the wave vector components along *x*- and *y*-axes, while *ε*_*x*,eff_ and *ε*_*y*,eff_ denote the permittivity along the two axes. Based on the aforementioned theory of waveguide plasma, *ε*_*x*,eff_ (*ε*_*y*,eff_) can be evaluated by *ε*_*x*_ − *c* ^2^/4*f* ^2^ *h* ^2^ (*ε*_*y*_ − *c* ^2^/4*f* ^2^ *h* ^2^), where *c* denote the light speed in a vacuum, *f* is the frequency under consideration, and *ε*_*x*_ (*ε*_*y*_) is the effective permittivity of the layered medium. In the work [40], via properly choosing the high *h* of the parallel-plate waveguide, one can obtain a negative zero *ε*_*y*,eff_ while keeping *ε*_*x*,eff_ a positive value. In this way, the ratio between the permittivity along *x*- and *y*-axes, measuring the anisotropy of the permittivity, approaches infinity, which thus results in an extremely “flat” hyperbolic equal frequency contour, as shown in the lower panel of Figure 2a. The waveguide-emulated hyperbolic medium was also experimentally verified for the field confinement in a deep-subwavelength scale [42] and negative refraction of wave [43].

Enhanced optical non-reciprocity was demonstrated in the work [44] via the waveguide merged with magneto-optical materials, as shown in Figure 2b. Consider a rectangular waveguide filled by a magneto-optical material, whose effective permittivity is given by a rank-two tensor:

(1) $$\bar{\bar{\epsilon }}=\left(\begin{array}{lll} \epsilon_{\text{mo}} & -\text{i}\alpha & \text{0}\\ \text{i}\alpha & \epsilon_{\text{mo}} & \text{0}\\ \text{0} & \text{0} & \epsilon_{⊥} \end{array}\right)\text{,}$$

where *ε*_mo_ and *ε*_⊥_ are the diagonal components of the permittivity tensor, and the effect of structural dispersion of the waveguide is encapsulated in *ε*_mo_. The off-diagonal component *α* is responsible for the strength of magneto-optical activity, which is typically a small value (∼10^−2^) in the optical region. It is well known that magneto-optical materials support the asymmetric propagation of circularly polarized waves. As solved from Maxwell’s equations, wave vectors of forward (+) and backward (−) propagating left-handed circular polarized (LCP) waves are given by *k* ^+^_LCP_ = (*ω*/*c*) $\sqrt{\epsilon_{mo}-\alpha }$ and *k* ^−^_LCP_ = (*ω*/*c*) $\sqrt{\epsilon_{mo}+\alpha }$ , while wave vectors of forward and backward propagating right-handed circular polarized (RCP) waves are, respectively, given by *k* ^+^_RCP_ = (*ω*/*c*) $\sqrt{\epsilon_{mo}+\alpha }$ and *k* ^−^_RCP_ = (*ω*/*c*) $\sqrt{\epsilon_{mo}-\alpha }$ . As proposed in Ref. [44], by modifying the cross-sectional size of the waveguide, one can make effective permittivity *ε*_mo_ close to zero. In this manner, the waveguide structure is transparent for the forward propagating RCP and backward propagating LCP waves whose wave vectors are real, while being opaque for the backward propagating RCP and forward propagating LCP waves with purely imaginary wave vectors. By this strategy, without modification to material properties, the “effective” non-reciprocity of the whole structure can be substantially enhanced.

Inspired by the energy squeezing effect found in narrow zero-permittivity channels, the seminal work by Argyropoulos et al. [45] presented an efficient solution to enhance Kerr (third-order) nonlinearities. As shown in Figure 2c, a nonlinear metamaterial slab is constructed by embedding nonlinear optical materials with cross-sectional areas of *w* × *t* into a silver screen. The spacing of rectangular apertures filled with nonlinear materials are *a* and *b*, respectively, along the *y*- and *x*-directions. Inserting this nonlinear metamaterial slab into a waveguide channel operating near the cut-off frequency, an electric field enhancement factor of *a* × *b*/(*w* × *t*) can be obtained, as a result of the power conservation. In this work [45], the electric field in the aperture is increased by more than 16 times compared with the incidence. Consequently, the Kerr nonlinear response, which scales linearly with the third order of the electric field, can be substantially enhanced by this strategy. The highly uniform electric field enhancement on the waveguide-emulated plasmonic resonance proves to be more favorable for boosting nonlinearity effects, as compared with the non-uniform electric field enhancement in Fabry-Perot resonances. Furthermore, the bistable operation of this nonlinear plasmonic waveguide channel is demonstrated, which is promising for applications in optical switches with low threshold intensities, optical memories, self-tunable devices, and so on.

At the end of this section, we introduce the implication of waveguide effective plasmonics for several interesting topics in quantum optics. The work by Sokhoyan and Atwater [46] theoretically investigated the quantum optical properties of a dipole emitter interacted with a nanoscale waveguide, via the approach of dyadic Green’s function-based field quantization method. It is demonstrated that, when the waveguide operates near the cut-off frequency, i.e., its effective plasma frequency, the spontaneous emission, and photonic lamb-shift effect are substantially enhanced. The enhanced spontaneous emission was experimentally verified via the use of a SiO_2_ waveguide with silver cladding [47], which operates at the cut-off frequency to emulate the plasmonic resonance. The work by Fleury and Alù [48] discussed the boost of the collective spontaneous emission, termed “superradiance,” of a collection of quantum emitters embedded in a waveguide-emulated plasmonic medium. When superradiance happens, the radiation intensity is dramatically increased and proportional to the square of the number of emitters. The perspective view and side view of the waveguide structure as the plasmonic host is shown in the upper panel of Figure 2d. To shed light on the superradiance enhancement, the Purcell factor, defined as the spontaneous emission rate over its value in a vacuum, is calculated and plotted in the bottom panel of Figure 2d. The result shown in Figure 2d is a function of both frequency and position of the emitter along the waveguide channel. As is clearly seen, the Purcell factor is uniformly enhanced along the channel near 400 THz, the cut-off frequency of the waveguide. As the magnitude and phase of the electric field along the waveguide plasmonic channel is highly uniform, lights emitted from quantum sources located in arbitrarily positions are highly coherent, which essentially contribute to the enhanced superradiance. More recently, the platforms of plasmonic waveguides have been developed to observe quantum entanglement [49, 50], and hybrid surface plasmon modes [51], which would find promising applications in the thriving fields of quantum information processing and quantum computation.

### 2.3 Photonic doping and metatronics

Photonic doping is one of the most fascinating applications in plasmonics, more specifically, in epsilon-near-zero (ENZ) materials. Originally, doping is a concept in the semiconductor field and means introducing impurities to materials aiming to control the macroscopic electric, magnetic, or optical properties. This concept has earned success in semiconductors. For example, doping makes it possible to tailor the conductivity, which is critical in the development of transistors and diodes. In principle, this concept of semiconductor doping could be transplanted to other areas, such as macroscopic photonics and metamaterials. Generally, a macroscopic dopant cannot construct or modify a homogenous body in macroscopy. However, the dopant in ENZ media has the capacity to tune the global permeability of the host media, which shows a new approach to controlling permeability and realizing special material responses. In fact, the ENZ host with defects doped has shown its applications, such as constructing an equivalent perfect magnetic conductor [52], object cloaking [53], and controlling reflection or transmission [54], etc.

**Figure 3:**
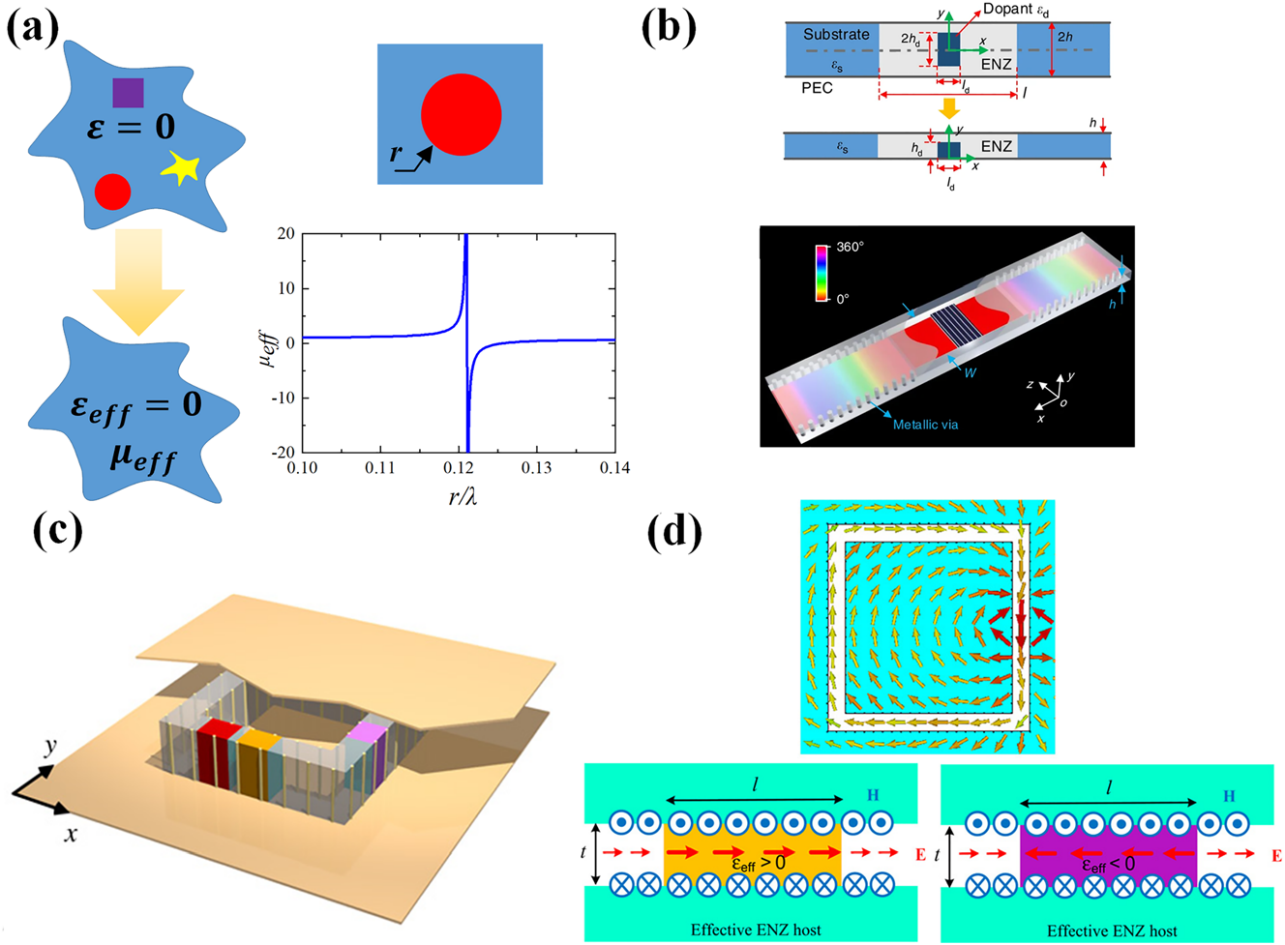
(a) Concept of photonic doping and the effective permeability; (b) substrate-integrated photonic doping [56]; licensed under the Creative Commons Attribution 4.0 International. (c) Conceptual structure of a waveguide metatronic system; (d) sketches of the D-dot wire, capacity and inductor elements [26]; (c) and (d) are reproduced with permission. Copyright 2016, AAAS.

The detailed principle of 2D photonic doping is displayed in Figure 3a. In 2D situations, dielectric particles immersed in the ENZ media behave as dopants, which are able to tune the universal permeability of the media [25]. In another word, the doped ENZ media behaves as a homogenous dielectric with a permittivity of zero and effective permeability of *μ*_eff_. The effective permeability *μ*_eff_ = 1 + Σ_d_ (∫∫*ψ*_d_(*r*)d*s* − *A*_d_)/*A*, where *A*_d_ is the area of the *d*th dopant, *A* is the area of the total ENZ media, and *ψ*_d_(*r*) is the magnetic field distributions inside the dopant, with the normalization value of 1 on the boundary. This expression implies that the equivalence is independent of the geometry of the ENZ host and the position of the photonic dopants, as for the ENZ region with permittivity of zero is equivalent to a point. As an instance, a dielectric rod with radius *r*, *ε*_d_ = 10, and *μ*_d_ = 1 is doped in the ENZ host with the area of 0.5*λ*_0_^2^. The field distributions in the rod can be obtained as *ψ*_d_(*r*) = *J*_0_(*k*_d_ *r*)/*J*_1_(*k*_d_ *r*). Then the effective permeability varying with the radius is plotted in Figure 3a. It is evident that the permeability could be arbitrarily tuned via adjusting the radius of the dopant. In particular, the effective permeability goes to infinity at the resonance frequency of the dopant, and the doped ENZ media is identical to a perfect magnetic conductor (PMC). Above resonance, there is a point at which the effective permeability equals zero.

Based on the principle of waveguide effective plasmonics, the 2D ENZ media could be effectively constructed, and various applications of photonic doping are realized in the waveguide platform. In [55], the photonic doping in waveguides is adopted to tune the global property of the ENZ host, and the spectral response is reconfigurable from PMC to EMNZ via adjusting the immersed particles, such as microfluidics to changing permittivity and mechanic actuators to rescale the dopant geometry. Another work adopting photonic doping in waveguides realizes the control of dipole emission and scattering [30]. When the dipole with spontaneous emission is embedded in the effective EMNZ media, the radiation gets significant enhancement with the enhancement rate varying with the relative position between the dipole and the dopant. In addition, the radiated power is always divided equally to the output ports as long as they have equal cross-sections.

As important progress, the technique SIW is introduced as the planar platform of effective photonic doping, promising high integration and potential for massive production [56]. As illustrated in Figure 3b, a square dielectric is doped in the waveguide effective ENZ region for the convenience of dopant fabrication. As for a square dopant, the effective permeability has a similar dispersion with the circular dopant and is able to realize the same property, though the specific expression is more complicated. When the dopant is positioned at the symmetry plane of the ENZ region, benefited from the symmetry of the electromagnetic fields, the total dimension can be reduced by one half with a perfect electric conductor replaced on the symmetry plane. This substrate-integrated photonic doping has validated the EMNZ property and applications in printed circuit board designs. Further, based on the integrated waveguide, a general impedance matching is realized through photonic doping [57]. The transmission property and bandwidth can be simply manipulated by tuning the dopant to optimally guide power regardless of the type of the load. With the advantages above, this scheme of general impedance matching would gain various applications.

Another application benefiting from the waveguide effective plasmonics is metatronics. Metatronics is a concept originating in optics, providing lumped nanocircuitry at optical frequency inspired by metamaterials [58, 59]. Generally, circuits with lumped elements are applied at the relatively low frequency with scales electrically small, while devices in optics are usually with large dimensions compared with the operating wavelength. Researches from plasmonics science have demonstrated the interaction of electromagnetic wave and plasmonic nanoparticles, which is in the sub-wavelength scale. This new phenomenon leads to associations between plasmonic nanoparticles and lumped circuit elements. In fact, it is proved that nanoparticles with dimensions much smaller than wavelength can be treated as lumped nanoelements, such as nanoinductors, nanocapacitors, or nanoresistors, thus constructing the concept of metatronics with the paradigm in the traditional lumped circuits [58]. The nanoparticles have been modeled in the plane-wave background as a parallel circuits element and the property is determined by the permittivity of the nanoparticles. To be specific, for the permittivity, the positive real part represents lumped capacitor while the negative real part means lumped inductor, in addition, the existence of the imaginary part introduces loss in the nanoparticles and is modeled as nanoresistors. The exact value of the lumped element is determined by the permittivity and the geometry of the nanoparticles. As the concept is expanded to multi-nanoparticles, various functional lumped circuits can be realized. Along the incident wave the lumped elements are connected by the ENZ, guaranteeing the independence of the individual element, then the circuits can be constructed as the scheme of lumped circuits [59]. Linking the two fields of electronics and nano-optics, the concept of metatronics has provided another paradigm for nanoelectronics and nanophotonics.

For practical designs of the metatronics, the materials with negative and zero index can be realized by plasmonic materials. However, natural plasmonic materials have specific dispersion only in optical frequency and are with non-negligible loss. Waveguide effective plasmonics provides an alternative to plasmonic materials. In Ref. [26], the concept of waveguide metatronics is proposed as an enhanced form of optical metatronics based on the dispersion property of waveguide, and the circuit elements in waveguide implementation are systematically investigated. As illustrated in Figure 3c, the total structure is constructed in the parallel-plate waveguide operating in TE_10_ mode at exactly the cut-off frequency, thus leading to an effective ENZ host background. Then, the region of the circuits is filled with the dielectric with the effective permittivity equal to one, forming an effective channel in the ENZ media as a D-dot wire. As shown in Figure 3d, the displacement current in the D-dot wire can be written as **J** = −i*ωε*_eff_ **E**, therefore the displacement current can be confined in the D-dot wire, which is similar with the conductive wires, while in the ENZ background, we have effective permittivity equal to zero. The slabs inserted in the D-dot wire to different permittivity represent the lumped inductor or capacitor, which is depicted in Figure 3d. As mentioned above, the slab with positive effective permittivity acts as a capacitor, while negative effective permittivity signifies an effective inductor. In this situation, for a slab of length *l* and width *t*, the impedance is *Z* = *l*/(−*iωtε*_eff_), in another word, we have *C* = *tε*_eff_/*l*, and *L* = −*l*/(*ω* ^*2*^ *tε*_eff_), which is consistent with the relations for inductor and capacitor. A multi-order metatronic filter is realized in the microwave domain in Ref. [60]. It is known that paralleling inductors in the circuits are high-pass filters, thus in this research, the host medium has an effective permittivity of 1 and the inductive elements have effective permittivity of −4.15. Note that the materials adopted are all normal materials at microwave frequency, and the metatronics are realized by waveguide effective plasmonics. The design paradigm of the metatronics has been sufficiently verified through the different ordered high-pass filters. Finally, Ref. [61] has completed the experimental validation of the metatronics in the platform of SIW, which is fabricated by the printed circuits board, exhibiting the low-loss realization and the potential in microwave and terahertz applications.

## 3 Transmission structures

Transmission structures are indispensable parts of optical and microwave systems. Due to the unique characteristics of plasmonics in nanoparticles, lots of interesting transmission structures based on plasmonics are investigated for novel functionalities in the past decades. Considering the further integration of optical devices, plasmon waveguides are proposed for the transmission of light. A metal nanoparticle waveguide composed of closely spaced metal nanoparticles is designed to allow the coherent propagation of light energy in sub-wavelength guiding structures, by utilizing the coupled plasmonic modes between adjacent particles [12]. By applying the silver rods as metal nanoparticles, a plasmonic waveguide is fabricated to observe the localized excitation [62]. For further investigation, the numerical analysis on the surface plasmon characteristics of plasmonic waveguides is systematically studied and presents new routes for integrated optoelectronic circuits [63, 64]. Besides the conventional insulator-metal-insulator (IMI) waveguide, the metal-insulator-metal (MIM) plasmonic waveguide is also widely discussed, showing the ability to reduce the volumes and minimize the field decay [65]. To further extend the application range of plasmonic-based transmission structures, flexible plasmonics [66] has drawn attention recently, offering the capability to propagate energy on nonplanar devices (e.g., flexible curved surfaces and flexible bending waveguides). Various materials and approaches are utilized to realize the flexible control of waves. For example, as demonstrated in Ref. [66], transmission structures on flexible curved surfaces are obtained by utilizing the transformation optics in graphene; epsilon-near-zero (ENZ) materials are investigated to realize the tunneling of energy through devices with arbitrary bending structures or subwavelength channels [67], [68], [69].

The structures mentioned above are based on natural plasmonic materials, dissipative losses may deteriorate the transmission rate of energy in the devices. Therefore, the waveguide effective plasmonics attracts wide interests and provides potential pathways to design low-loss and highly-integrated transmission structures [23], especially for microwave and terahertz regimes. The waveguide effective plasmonics is realized by utilizing the structural dispersion of waveguide, and the effective negative permittivity can be obtained with non-dispersive and low-loss dielectrics with positive permittivity [70]. Based on this concept, effective localized surface plasmons are studied to design resonators with high *Q*-value in deep subwavelength scales, reproducing the characteristics of optical localized surface plasmons [71, 72].

**Figure 4:**
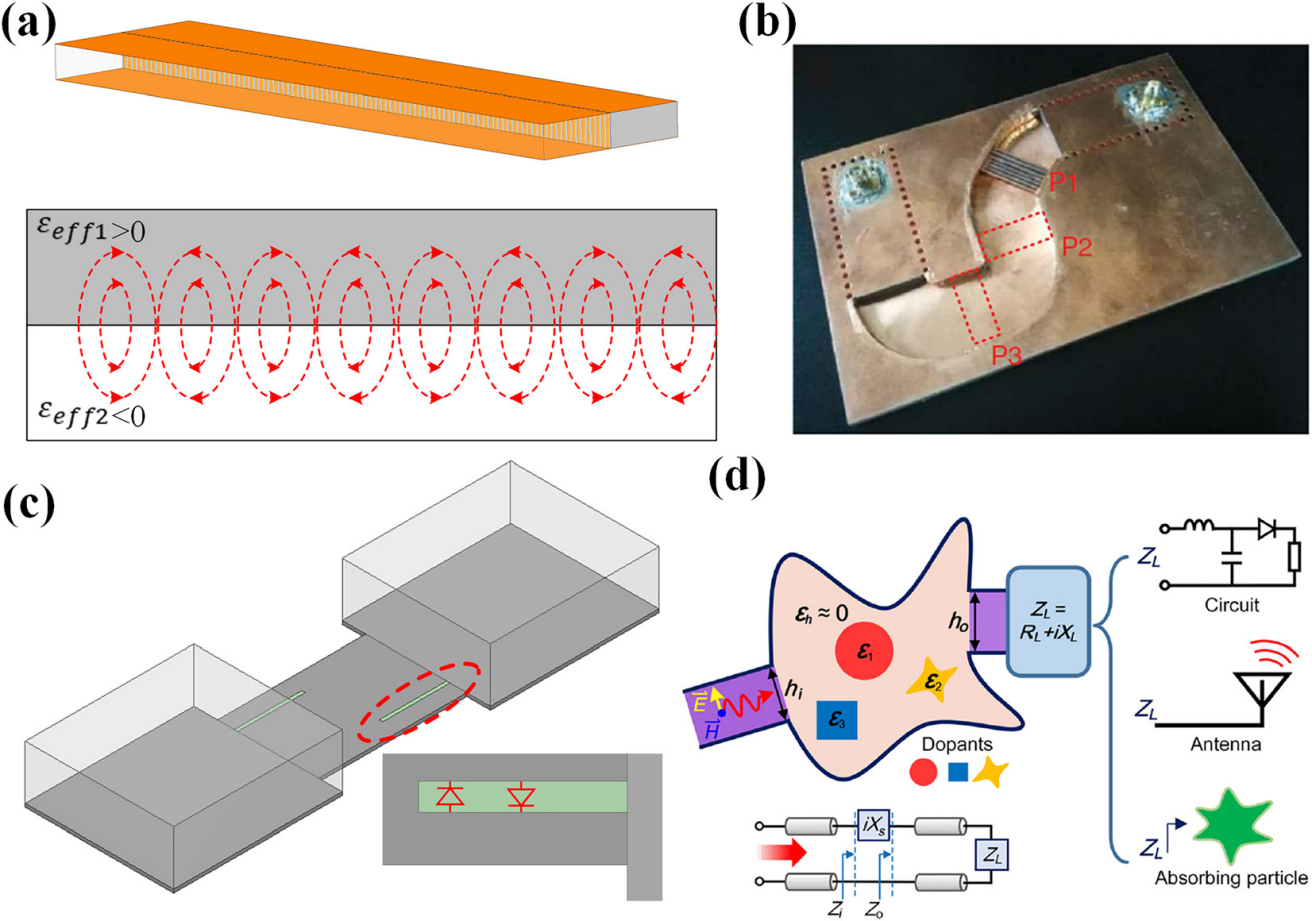
(a) Surface plasmon polaritons in waveguide at microwave frequency [73]; (b) deformable waveguide transmission line made of EMNZ [56]; licensed under the Creative Commons Attribution 4.0 International. (c) frequency-tunable waveguide channel [80]; (d) general impedance-matching network [57]; reproduced with permission. Copyright 2020, American Physical Society.

As an essential type of transmission structure in optical devices, SPPs can also be mimicked at the interface of two conventional dielectric materials (i.e., positive permittivity) inside a parallel-plate waveguide, avoiding the losses from natural negative-permittivity materials [73]. As depicted in Figure 4a, the waveguide region *y* < 0 is filled with low-loss material with positive relative permittivity of *ε*_act1_ = 2.2, and the waveguide region *y* > 0 is filled with air with relative permittivity of *ε*_act2_ = 1. According to waveguide dispersion, the effective permittivity of these two regions are *ε*_eff1_ = 0.45 > 0 and *ε*_eff2_ = 0.45 < 0, which are determined by the dimensions of the waveguide and the operating frequency. The effective permittivities of two regions satisfy *ε*_eff1_ *ε*_eff2_ < 0 and thus, their interface supports the propagation mode of SPP. Experimental verifications are carried out at microwave frequencies and the measured results demonstrate that the propagation length of waveguide effective SPP is much longer than real SPP (i.e., utilizing negative-permittivity plasmonic materials). It is proved in Ref. [74] that effective SPP is applicable for both double-layered and multilayer systems, and can be supported by heterostructures (i.e., IMI and MIM in optical frequency). In Refs. [29, 75], the variation of propagation lengths is systemically demonstrated when characteristics of two media or geometrical parameters of waveguides are changed. Due to the low-loss and high-integration properties of effective SPP, it is utilized in various interesting investigations (e.g., broadband wave harvesting [76] and frequency splitter [77]). For transmission structures based on waveguide effective SPP, the electromagnetic wave usually propagates along the interface of two media. Recently, a new kind of transmission structure, in which the waveguide operates around the cut-off frequency of TE_10_ mode, is proposed in Ref. [56] by constructing a doped ENZ cavity, as shown in Figure 4b. The ENZ medium in this cavity is realized by waveguide effective plasmonics. Analytical and experimental results demonstrate that total transmission can be always realized by this structure, no matter the shape of the waveguide is deformed, exhibiting huge applicable potential in devices with huge geometrical discontinuities.

Inspired from the concept of wave supercoupling of natural plasmonic materials, the waveguide effective structure for supercoupling can provide a feasible approach to transmit energy within discontinuous structures. The concept of waveguide effective supercoupling is first verified by Engheta through an experiment in microwave frequency. The total transmission effect is realized through a narrow waveguide channel which exhibits effective zero-permittivity properties, without the utilization of resonant inclusions [22]. Moreover, effective epsilon-and-mu-near-zero (EMNZ) supercoupling is proposed and enables the tunneling effect between two separated emitters with arbitrary distance and orientation [78]. Researchers also extended the waveguide effective supercoupling effect to design spoof SPPs [79], which is a useful type of transmission structure and with similar characteristics with SPPs. To broaden the operating bandwidth of supercoupling transmission, two longitudinal slots are etched on the top surface of the effective ENZ channel as shown in Figure 4c. Two p-i-n diodes are placed across the slots and the supercoupling frequency will vary with the length of slots (i.e., the off/on state of diodes). Experimental results indicate a tuning range of 1.06 GHz [80]. Another feasible approach to realize effective ENZ supercoupling control in a wide bandwidth is proposed by utilizing a reconfigurable channel. In Ref. [81], a metasurface structure composed of periodically positioned blind vias is inserted in the channel and the frequency is controlled by mechanically tuning the inserting depth.

Interesting properties of waveguide effective transmission structure pave new ways to design devices with different functionalities. As depicted in Figure 4d, the effective doped ENZ transmission line is utilized to realize a general impedance matching network [57]. Reflected waves can be removed by tuning the dopants’ properties. This matching network can be applied in various scenarios of circuits, antennas, and absorbing particles. A three-dimensional plasmonic cloaking is realized by effective negative permittivity on a finite cylinder core [82]. The scattering suppression is strong in wideband range and robust for the directions of illumination and observation. In Ref. [83], by connecting the ENZ cavity with multiple waveguides, a novel design of N-port split power dividers is designed. The amplitude and phase of each port can be precisely determined by the dimensions of the output waveguide. Furthermore, a wideband waveguide crossover is proposed by superposing the ENZ and Fabry–Perot modes, providing a feasible approach for waveguide-integrated devices [84].

## 4 Filters and sensors

Apart from mentioned transmission structures to realize high energy-transmission rates, plasmonics is also widely engaged in other kinds of wave-manipulation devices, i.e., filters for controlling the attenuation of undesired signals [85] and sensors for detection of desired signals [86]. By adding the asymmetrical multiple-teeth-shaped structure in the plasmonic waveguide, an optical filter is realized with a narrow passband and the spectrum can be tuned by the dimensions of the asymmetrical structure [87]. A planar bandpass filter is fabricated on the platform of monolayer graphene. The graphene plasmonic structure (i.e., graphene ribbons and rings) is utilized to electrically control the transmission peak within mid-infrared frequencies [88]. Recently, as a novel concept, optical metatronics indicates that the subwavelength nanoparticles with positive/negative permittivities can behave as “lumped” capacitors/inductors and conventional lumped circuit theory can be transferred to the design of optical devices. Therefore, different filters with low-pass, high-pass, band-pass, and band-stop properties are respectively presented in Ref. [89] by properly combining different plasmonic slabs (negative permittivities) and dielectric slabs (positive permittivities). Plasmonic sensors operating with different mechanisms are proposed for various multidisciplinary research fields. A plasmonic device composed of a gold mirror and a 2-D periodical gold array is fabricated in Ref. [90], simultaneously exhibiting the properties of an infrared perfect absorber and a localized surface plasmon resonance (LSPR) sensor. Another LSPR sensor with high sensitivity is realized through a complementary planar metamaterial [91], which contains two types of optical antennas (i.e., bright dipole and dark quadrupole antennas).

**Figure 5:**
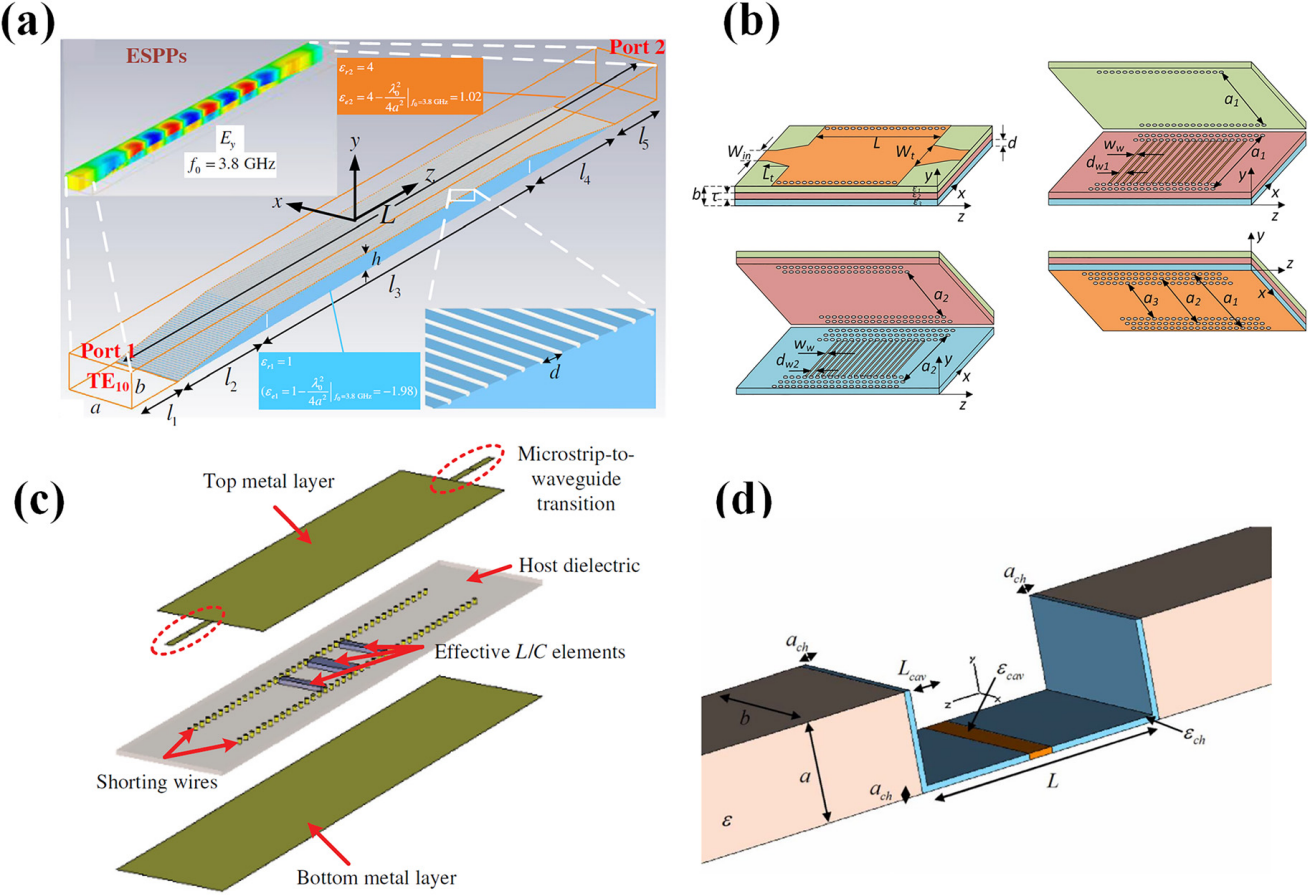
(a) Model-dispersion-induced SPP filter [28]; reproduced with permission. Copyright 2017, American Physical Society. (b) Dual-band band-pass filters based on surface plasmon polaritons-like propagation [92]; licensed under the Creative Commons Attribution 4.0 International. (c) Lumped filter circuits based on metatronics [61]; reproduced with permission. Copyright 2018, American Physical Society. (d) Permittivity sensor in ENZ channels [96]; reproduced with permission. Copyright 2008, American Physical Society.

The above-mentioned plasmonic filters and sensors greatly facilitate the development of nanodevices in optical frequencies [34]. Here, the concept of waveguide effective plasmonics opens up a feasible pathway to further extend plasmonic filters and sensors’ applicable range to microwave and terahertz frequencies. Some representative researches are described in detail in this section. As depicted in Figure 5a, based on the concept of waveguide effective plasmonics, the effective surface plasmon polaritons (ESPP) is observed in the rectangular waveguide and propagates at the interface between two dielectrics with different positive permittivities. Subsequently, transverse-electric modes (e.g., TE_10_ mode, and TE_20_ mode) in the rectangular waveguide are utilized to induce the ESPP within a passband between 3.28 GHz and 4.1 GHz, by constructing a mode-conversion structure composed of two slopes and plenty of parallel thin metallic wires. Theoretical and numerical investigation on the passband are carried out and agree quite well, exhibiting that the proposed approach has the potential to be applied for the design of waveguide effective plasmonic filters [28]. Similarly, waveguide effective SPP is utilized to design dual-band band-pass filters in [92] to meet the requirement of multiple spectrums in the next-generation communication systems. Two frequency bands with zero-reflection properties are realized by inducing two distinct effective SPP propagations at the interface of a three-layer substrate integrated waveguide, as shown in Figure 5b. Three sub-SIW layers have different permittivities of filled dielectrics and geometrical dimensions respectively, providing enough degrees of freedom to arbitrarily tune the center frequencies of two passbands within a wide range of spectrums. Prototypes are fabricated with standard print-circuit-board (PCB) processing, exhibiting the merits of low cost and low profile. Then, researchers redesign these dual-band filters using the low-temperature co-fired ceramic (LTCC) technology, further improving fabrication accuracy, robustness, and integration [93]. Different from these three filters inspired by waveguide effective SPPs, waveguide effective plasmonic nanoparticles also provide a promising route to design effective plasmonic filters.

In previous sections, we have introduced that the concept of metatronics provides optical lumped elements to design nanocircuits [89]. In addition, the waveguide metatronics based on waveguide effective plasmonics provides an easy-fabrication platform to implement optical metatronic circuits in other frequencies (e.g., microwave and terahertz frequencies). A three-order high-pass filter based on waveguide metatronics [61] is illustrated in Figure 5c. It can be seen that three dielectric slabs are inserted inside the host dielectric of the substrate integrated waveguide. The actual relative permittivities of dielectric slabs and host dielectric are all positive. According to the structure dispersion of waveguides, the effective relative permittivities dielectrics can be calculated. If the inserted slab has a positive (negative) effective permittivity, it behaves as a lumped capacitive (inductive) element. Therefore, by placing numbers of dielectric slabs with different permittivities, effective L/C elements are created in waveguides and can be utilized to design waveguide circuits for different applications. Example circuits containing one, two, and three negative-effective-permittivity dielectric slabs are fabricated and investigated, respectively, and first-, second-, third-ordered high-pass filters are successfully realized. In Ref. [94], two series of dielectric slabs are periodically inserted inside the rectangular waveguide, and the effective relative permittivity of dielectric slabs 1 is negative and that of dielectric slab 2 is positive. This multilayer structure performs as an effective plasmonic filter, which enables the suppression of spatial harmonics. Then, this filter is utilized to realize the edge detection in terahertz frequencies, exhibiting merits of high contrast and subwavelength resolution up to 0.1*λ*.

On the other hand, sensors based on waveguide effective plasmonics attract attention recently, providing a potential approach to reproduce the excellent performance of optical plasmonic sensors (e.g., high selectivity and sensitivity). In the work [95], waveguide effective SPP propagation is induced through two stacked half-mode SIWs (effective relative permittivities of filled dielectrics have opposite signs), achieving a sharp zero-transmission spectrum in waveguide response. The frequency of zero-transmission point is highly sensitive to the relative permittivities of SIWs. Then, a microwave sensor is designed for the detection of relative permittivity. In this experiment, different fluids are injected into the microfluidic reservoir to mimic the permittivity variation of SIW, and detected results agree well with the actual permittivities. This work opens up a new route to design high-performance microwave sensors with the merits of plasmonics.

Besides the zero-transmission spectrums, the zero-reflection spectrums of some waveguide effective plasmonic devices can also be utilized to design microwave sensors. In Figure 5d, an ultranarrow waveguide effective ENZ channel is placed between the input and output rectangular waveguides. As stated in Section 3, the effective supercoupling effect is realized and there appears a sharp zero-reflection peak in the spectrum. The field within the narrow channel is enhanced dramatically and thus, a tiny variation on the dielectric permittivity of this channel will lead to a significant shift of the transmission peak. This phenomenon is exploited to design a sensor for the description of the permittivity perturbations (i.e., the orange region in Figure 5d in the ENZ channel [96]. An accurate model is constructed and closed-form expressions are derived to guide the engineering practice of this idea in Ref. [96]. Subsequently, a sensor containing a multilayered ENZ SIW channel is presented in Ref. [97], enabling the measurement of complex permittivity of dispersive materials at two frequencies. Experimental results indicate that this sensor based on waveguide effective plasmonics has a high sensitive and low sensing error.

## 5 Antennas and lenses

Plasmonics is also widely exploited in the design of plasmonic antennas and metasurfaces to realize the conversion between localized energy and free propagation radiation, the manipulation of the wavefront in free space, and the steering of a beam, respectively [98]. In Ref. [99], the fundamental operating principles and advantages of plasmonic antennas in optical frequencies are described in detail, indicating that plasmonic antennas provide an unprecedented approach to control the light-matter interactions and can be applied in optical devices. For example, one of the interesting applications is reported in Ref. [100] and plasmonic antennas are utilized to improve the performance of photodetection. Plasmonic nanoantennas are also usually utilized as the elements of optical metasurfaces for different functionalities. A plasmonic nanoantenna array is investigated in Ref. [101] to manipulate the wavefront of light, leading to the unique light-bending phenomenon within a wide frequency region. Plasmonic nanoparticles are adopted to form a Yagi-Uda antenna in Ref. [102], and the nanoantenna can dynamically tune the radiation performance via plasma excitation. In Ref. [103], planar and ultra-thin metalenses composed of individual Babinet-inverted nanoantennas are proposed to control the focal length of light and separate light at different wavelengths. To widen the applicability of plasmonic metasurfaces, dielectric gradient metasurface optical elements based on Si nanobeam antennas are proposed to realize ultrathin gratings, axicons, and lenses, exhibiting excellent performances and high integration level with semiconductor devices [104]. Apart from these plasmonic antennas based on metasurfaces, lumped metatronic nanoparticles are also adopted for optical antennas [105]. In Ref. [106], the characteristics of plasmonic nanodipoles are explored, demonstrating that the input impedance and radiation performance can be optimized by engineering the nanoparticle elements. This work extends the design methodology of RF antennas to optical regimes. Correspondingly, the design methodology and operating properties of optical antennas can also be extended to the design of RF antennas by exploiting the concept of waveguide effective plasmonics. Next, we will discuss some new designs of waveguide effective plasmonic antennas.

ENZ material has attracted wide attention in the investigation of optical plasmonics due to its unique properties that are different from traditional materials. According to waveguide effective plasmonics, effective ENZ material can be realized in the waveguide by properly designing the structural parameters of the waveguide. Within the effective ENZ material, the phase velocity and wavelength of an electromagnetic wave are both infinite, demonstrating that the spectrum response is independent of the geometry and shape. Following this counter-intuitive phenomenon, various geometry-independent ENZ antennas are explored to achieve exotic functions and characteristics.

**Figure 6:**
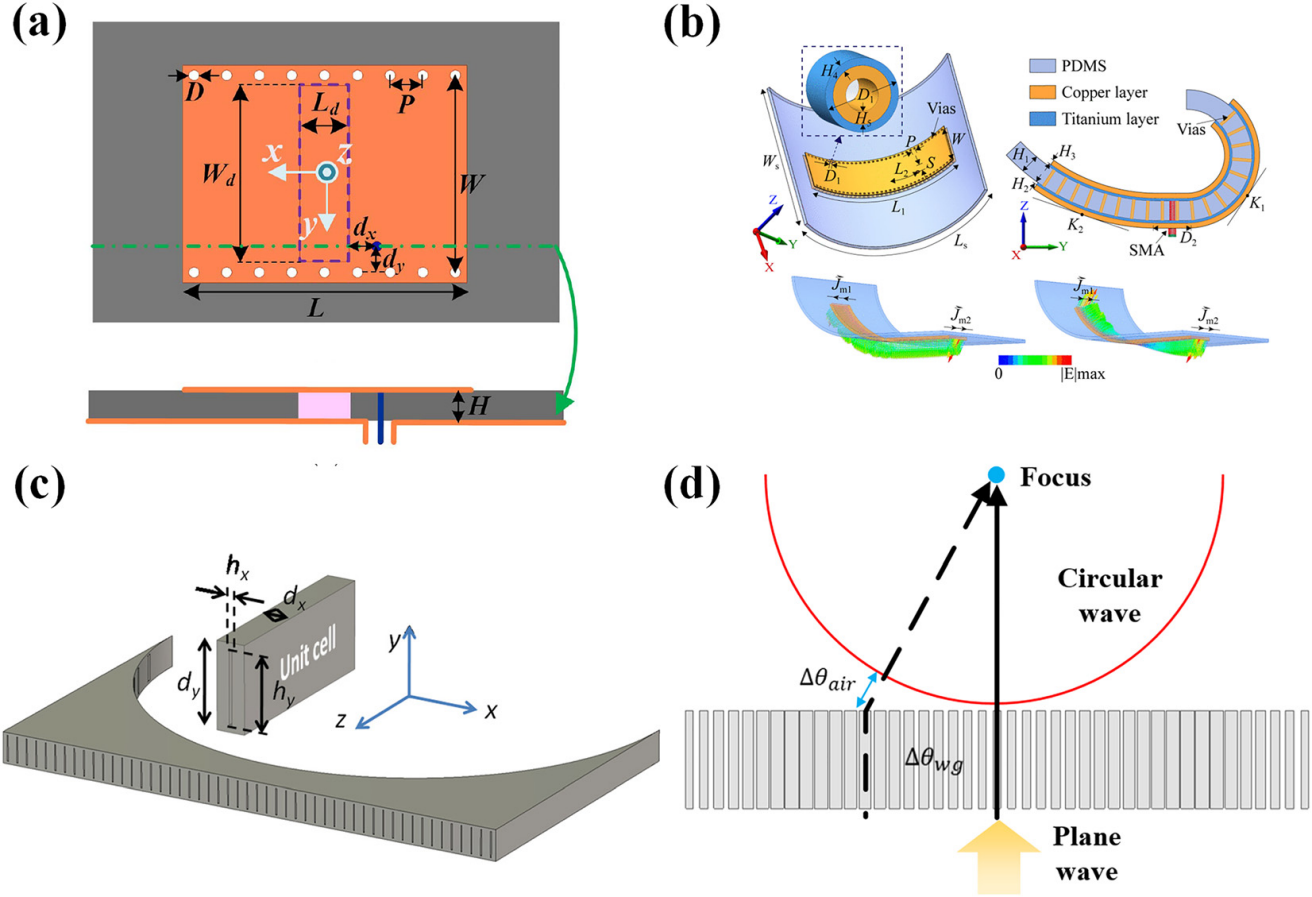
(a) A doped ENZ antenna [107]; reproduced with permission. Copyright 2020, IEEE. (b) ENZ-inspired deformable antenna [110]; reproduced with permission. Copyright 2020, IEEE. (c) Waveguide-based ENZ lens realizing the furrier transformation [112]; reproduced with permission. Copyright 2012, American Physical Society. (d) An epsilon-near-zero graded-index converging lens with planar faces [113].

In Ref. [27], the effective ENZ mode is obtained at the transverse cut-off mode of the rectangular waveguide. The waveguide has two open ends that can be regarded as two radiation apertures. The phases and amplitudes of E-fields on these two apertures are the same, leading to a pair of antiphase magnetic currents. With the variation of waveguide length, the operating frequency of the effective ENZ mode keeps unchanged, but the distance between two magnetic currents is varied. Therefore, the angles between two beams can be freely tuned at the same resonant frequency, exhibiting the length-independent property of this type of antenna. A prototype with a planar-curved structure is investigated to demonstrate the shape-independent property of waveguide effective ENZ materials. After curving the waveguide, two magnetic currents are in phase and a broadside radiation pattern is achieved. Then, another methodology to construct in-phase magnetic currents for broadband radiation is proposed by exploiting the concept of photonic doping [107]. As shown in Figure 6a, a ceramic block with extremely high relative permittivity is “doped” into the SIW. Due to the high permittivity, the block has an electrical distance of half the wavelength and a narrow physical distance at the operating frequency. The E-field phase is inversed by 180° when the wave propagates through the block, and thus the E-fields on the two open ends are antiphase. According to the calculation formula of effective magnetic current ${\vec{J}}_{m}=-2\hat{n}\times \vec{E}$ , antiphase normal directions and antiphase E-fields result in the in-phase magnetic current. Therefore, the broadside radiation can be realized without curving the SIW structure. Theoretical and experimental results indicate that the operating frequency is independent of the length of the waveguide and the doped position of the ceramic block. On the basis of the antenna in Ref. [27], a series-fed two-element antenna array is proposed in Ref. [108]. A half-wavelength microstrip line is exploited to connect two elements and provides 180° phase shift. This array achieves a four-beam radiation pattern. The realized gains and beam directions can be manipulated at the same frequency by tuning the length of the element. Subsequently, considering the requirement on polarization diversity in wireless communication systems, length-irrelevant dual-polarized effective ENZ antennas are presented in Ref. [109]. Different from photonic doping in Ref. [27], the in-phase magnetic currents on two open ends are achieved by a slot etched on the bottom metal of the waveguide. Two SIW ENZ antennas are placed orthogonally and fed through a cross-shaped slot, leading to the dual-polarized property. A backed cavity is assembled under the waveguide to suppress the back radiation of cross slots. Two prototypes operating at the same frequency are fabricated with different waveguide lengths for different applicable scenarios: one achieves a radiation pattern without sidelobes; another one achieves a radiation pattern with a high realized gain of 10.56 dBi. This literature further demonstrates that the waveguide effective ENZ antennas can be realized on flexible substrates like PDMS and LCP, and works well when the flexible substrates are bent or stretched. A flexible effective ENZ antenna is fabricated on the PDMS substrate and its performance is experimentally validated [110], as shown in Figure 6b. Different bent states are investigated to illustrate that this antenna with strong robustness, showing potential applications in wearable/body-centric integrated systems. In Ref. [111], a compact multiband antenna is achieved based on the ultra-narrow waveguide effective ENZ channel. Benefited from the exotic property of ENZ, the design of the antenna is flexible and tunable with a simple coaxial cable feeding.

Waveguide effective plasmonics can be exploited to design microwave/terahertz lenses for the manipulate of the electromagnetic wave in free space. As depicted in Figure 6c, an array of waveguides whose filled dielectric has the effective relative permittivity of near zero is utilized to construct a plano-concave ENZ lens [112]. A spatial Fourier transformation is realized on the focal plane and the impinging wave can be focused onto a single point. A similar idea is also used in the design of a terahertz ENZ graded-index converging lens [113], as shown in Figure 6d. The lens is composed of several waveguides operating slightly above the cut-off frequency. Different from the structure in Figure 6c, these waveguides in Figure 6d have the same length. The cross-sectional sizes of waveguides are different, enabling the individual manipulation of the propagation constant of each waveguide. These two lenses based on waveguide effective ENZ material exhibit merits of low loss and precise phase control. Another 2-D lens formed by the waveguide effective ENZ channel is designed in Ref. [114]. An anomalous matching of nearby sources is adopted and simply patterning tailoring is realized, showing exciting properties of the novel lens. In Ref. [115], a waveguide effective superlens is proposed by periodically filling two dielectrics with different positive permittivities in a rectangular waveguide. The lens successfully enhances the evanescent waves and improves the imaging resolution.

## 6 Conclusion and outlook

The waveguide effective plasmonics has been experiencing rapid development and has attracted researches in various fields for diverse applications, as this concept has connected electronics and optics to inspire innovations. In this review, we have first reviewed the concept of plasmonics and demonstrated the inherent loss in natural plasmonic materials, starting from that, the concept of waveguide effective plasmonics is introduced. Then, we have systematically analyzed the physical nature of structural dispersion and the concrete design methodology of plasmonics, exhibiting the key features of the waveguide effective plasmonics. Based on the waveguide effective plasmonics, exciting applications have been proposed, including classical plasmonic concepts, irregular material response, and novel microwave devices with plasmonics. It is undoubted the waveguide effective plasmonics is gradually emerging as a promising concept.

Besides the applications reviewed above, there still exist various functions for plasmonics. Waveguide effective plasmonics as a flexible and low-loss platform to realize plasmonics will continue to play an important role in introducing plasmonic properties to lower frequency, which would broaden the applications of plasmonics and inspire novel functions in microwave or terahertz frequencies. On the other hand, the SIW technique makes it possible to build highly integrated waveguide systems, and so as the waveguide effective plasmonics. Waveguide effective plasmonics in SIW can be integrated into a planar system by printed circuit board technique, silicon micromachining, or nano-fabrication, and remains the properties of low loss and low crosstalk, which is promising in massive-integrated waveguide systems, or even circuits on-chip.
